# Häusliche Gewalt und ihre psychischen Folgen während der COVID-19-Pandemie – Zentrale Befunde aus dem deutschsprachigen Raum

**DOI:** 10.1007/s00103-023-03747-8

**Published:** 2023-07-10

**Authors:** Annett Lotzin, Aleya Flechsenhar, Susan Garthus-Niegel, Anna Katharina Georg, Julia Holl, Leonie von Hülsen, Laura Kenntemich, Sören Kliem, Christoph Kröger, Judith T. Mack, Amera Mojahed, Sabine Nunius, Johanna Schröder, Katja Seitz, Alexandra von Thadden, Jana Volkert, Irina Zrnic Novakovic, Brigitte Lueger-Schuster

**Affiliations:** 1 Klinik und Poliklinik für Psychiatrie und Psychotherapie, Universitätsklinikum Hamburg Eppendorf, Martinistr. 52, 20246 Hamburg, Deutschland; 2 Institute for Clinical Psychology and Psychotherapy (ICPP), Department Psychologie, MSH Medical School Hamburg, Hamburg, Deutschland; 3 Lehrstuhl für Klinische Psychologie und Psychotherapie, Ludwig-Maximilians-Universität München, München, Deutschland; 4 Institut und Poliklinik für Arbeits- und Sozialmedizin, Medizinische Fakultät, Technische Universität Dresden, Dresden, Deutschland; 5 Institut für Psychosoziale Prävention, Zentrum für Psychosoziale Medizin, Universitätsklinikum Heidelberg, Heidelberg, Deutschland; 6 Fachbereich Sozialwesen, Ernst-Abbe-Hochschule Jena, University of Applied Sciences, Jena, Deutschland; 7 Department of Welfare, Ernst-Abbe-Hochschule Jena, University of Applied Sciences, Jena, Deutschland; 8 Abteilung Klinische Psychologie und Psychotherapie, Universität Hildesheim, Hildesheim, Deutschland; 9 SANU HEALTH, Erlangen, Deutschland; 10 Klinik für Allgemeine Psychiatrie, Zentrum für Psychosoziale Medizin, Universitätsklinikum Heidelberg, Heidelberg, Deutschland; 11 Fakultät Naturwissenschaften, MSB Medical School Hamburg, Berlin, Deutschland; 12 Institut für Klinische und Gesundheitspsychologie, Fakultät für Psychologie, Universität Wien, Wien, Österreich

**Keywords:** Gewalt, Trauma, Posttraumatische Belastungsstörung, COVID-19-Pandemie, Prävalenz, Risikofaktoren, Violence, Trauma, Post-traumatic stress disorder, COVID-19 pandemic, Prevalence, Risk factors

## Abstract

Die Auswirkungen traumatischer Erfahrungen auf die psychische Gesundheit während der COVID-19-Pandemie sind im deutschsprachigen Raum bisher unzureichend diskutiert worden. Vor diesem Hintergrund wurde im Auftrag der Deutschsprachigen Gesellschaft für Psychotraumatologie (DeGPT) eine Arbeitsgruppe aus wissenschaftlich und praktisch tätigen Fachkolleginnen und -kollegen gebildet. Ziel der Arbeitsgruppe war es, zentrale Forschungsbefunde zur Prävalenz von häuslicher Gewalt und damit einhergehende psychische Belastungen während der COVID-19-Pandemie im deutschsprachigen Raum zusammenzufassen und deren Implikationen zu diskutieren. Darüber hinaus sollten Zusammenhänge zwischen vorbestehenden Kindheitstraumata und psychischen Belastungen während der Pandemie beleuchtet werden. Hierzu wurde die vorliegende narrative Übersichtsarbeit erstellt.

Die Ergebnisse der durchgeführten Studien weisen auf hohe Prävalenzen häuslicher Gewalt während der COVID-19-Pandemie hin, die jedoch überwiegend den Prävalenzen vor der Pandemie entsprechen. Erwachsene, die während der Pandemie oder bereits in ihrer Kindheit oder Jugend interpersonaler Gewalt ausgesetzt waren, wiesen während der Pandemie eine erhöhte psychische Belastung im Vergleich zu Erwachsenen ohne Gewalterfahrungen auf. Eine Reihe an Faktoren (z. B. weibliches Geschlecht, geringe Sozialkontakte) erhöhten das Risiko für psychische Belastung und Symptome einer posttraumatischen Belastungsstörung während der Pandemie. Nach diesen Ergebnissen stellen Menschen mit aktuellen, aber auch zurückliegenden Gewalterfahrungen eine vulnerable Gruppe dar, die während einer Pandemie besondere Unterstützungsbedarfe aufweist.

## Abstract (en)

The impact of traumatic experiences on mental health during the COVID-19 pandemic has been insufficiently discussed in the German-speaking countries. Against this background, a working group of scientifically and clinically active colleagues was formed on behalf of the German-Speaking Society for Psychotraumatology (DeGPT). The aim of the working group was to summarize central research findings on the incidence of domestic violence and associated psychological distress during the COVID-19 pandemic in German-speaking countries and to discuss their implications. In addition, associations between pre-existing childhood trauma and psychological distress during the pandemic should be illuminated. The present narrative review was prepared for this purpose.

The results of the studies conducted indicate high prevalences of domestic violence during the COVID-19 pandemic, which, however, predominantly correspond to pre-pandemic prevalences. Adults with current or pre-existing interpersonal traumatic experiences during childhood or adolescence reported increased psychological distress during the pandemic compared with adults without such experiences. A number of risk factors (e.g., female gender, lower frequency of social contacts) increased the risk of psychological distress and posttraumatic stress disorder symptoms during the pandemic. According to these findings, people with current or past interpersonal trauma exposure represent a vulnerable group with special support needs during pandemic contexts.

## Einleitung

Auch wenn Infektionsschutzmaßnahmen während einer Pandemie aus virologischer Sicht notwendig sind, können sie für bestimmte Bevölkerungsgruppen körperliche und psychische Gesundheitsrisiken erhöhen. In diesem Zusammenhang wurde eine mögliche Zunahme an häuslicher Gewalt als eine unbeabsichtigte Folge der Infektionsschutzmaßnahmen diskutiert [1, 2]. Als mögliche Risikofaktoren gelten der Wegfall professioneller Unterstützungssysteme, die Einschränkung von Fluchtmöglichkeiten sowie finanzielle Einbußen [3]. Zu den psychischen Auswirkungen der Infektionsschutzmaßnahmen wurde während der COVID-19-Pandemie eine Reihe an europäischen Studien durchgeführt. Hierzu wurden psychische Belastungen, depressive oder Angstsymptome in der Allgemeinbevölkerung erfasst [4]. Die Häufigkeit erlebter häuslicher Gewalt während der Pandemie und damit einhergehende psychische Belastungen wurden weitaus seltener erforscht. Einzelne Studien aus dem europäischen Raum untersuchten psychische Belastungen während der Pandemie bei Erwachsenen, die in ihrer Kindheit häuslicher Gewalt ausgesetzt waren. Diese Studien wiesen auf eine erhöhte psychische Belastung während der Pandemie bei Erwachsenen mit vorbestehenden Kindheitstraumata hin [5, 6]. Biologische und soziale Risikofaktoren, wie z. B. finanzielle Belastung oder körperliche Erkrankung, standen bei von Kindheitstraumata Betroffenen mit einer erhöhten psychischen Belastung in Zusammenhang [7, 8].

Ein vertieftes Verständnis von Gewalt und anderen potenziellen Traumata während der COVID-19-Pandemie sowie deren Auswirkungen auf die psychische Gesundheit kann dazu beitragen, vulnerablen Gruppen in zukünftigen pandemischen Situationen oder anderen globalen Krisen einen besseren Schutz zu bieten. Für den deutschsprachigen Raum wurden die während der COVID-19-Pandemie durchgeführten Studien bislang noch nicht zusammengefasst. Vor diesem Hintergrund wurde im Auftrag der Deutschsprachigen Gesellschaft für Psychotraumatologie (DeGPT) eine Arbeitsgruppe gebildet (Autorinnen und Autoren dieses Beitrags). Von der Erstautorin wurden relevante publizierte und unpublizierte Studien in PubMed, PSYNDEX, Google und Studienregistern bis Juni 2022 identifiziert. Hierzu wurden Suchwörter zu Trauma und Gewalt eingesetzt, kombiniert mit Begriffen für die COVID-19-Pandemie sowie für den deutschen Sprachraum. Die Studienautorinnen und -autoren wurden kontaktiert und gebeten an der Arbeitsgruppe mitzuwirken. Die Arbeitsgruppe bestand entsprechend überwiegend aus Fachexpertinnen und -experten, die selbst Studien zu Traumata und Traumafolgestörungen während der COVID-19-Pandemie im deutschsprachigen Raum durchgeführt hatten, sowie aus im Bereich der Psychotraumatologie klinisch tätigen Psychotherapeutinnen. Die Arbeitsgruppe hatte das Ziel, Forschungsbefunde zu Prävalenzen von häuslicher Gewalt sowie Zusammenhänge zwischen früheren Kindheitstraumata und aktuellen psychischen Belastungen während der Pandemie darzustellen und deren Implikationen für Forschung und Praxis zu diskutieren. Hierzu wurde von der gebildeten Arbeitsgruppe die vorliegende narrative Übersichtsarbeit erstellt.

## Studienbefunde zur Prävalenz häuslicher Gewalt

Zum Themenbereich häusliche Gewalt wurden 4 Studien bzw. Sekundäranalysen in die Übersicht einbezogen (Tab. 1), die im Folgenden beschrieben werden.

**Table 1:** 

Studie	*N*	Messzeitpunkt	Studiendesign	Zielpopulation	Konstrukte und Messinstrumente	Hauptergebnisse
**Studien zu Prävalenzen häuslicher Gewalt während der COVID-19-Pandemie**						
Ebert & Steinert (2021) [9]	3818	T1 04–05/2020	Repräsentative Querschnittsstudie	Deutschland Frauen zw. 18–65 Jahren (*M* = 44,30, *SD* = 12,02) in Partnerschaft	Körperliche Gewalt und sexualisierte Gewalt: Selbstkonstruierter Fragebogen orientiert am WHO-Fragebogen zu häuslicher Gewalt	Prävalenzen: verbale Konflikte: 25,3 % (95 % KI 24,0–26,7); körperliche Konflikte: 3,1 % (95 % KI 2,5–3,6); emotionaler Missbrauch: Bedrohung: 3,8 % (95 % KI 3,2–4,4); eingesperrt 2,2 % (95 % KI 1,8–2,7); kontrolliert worden: 4,6 % (95 % KI 3,9–5,3); physische Gewalt: 1,5 % (95 % KI 2,1–5,1); nicht einvernehmlicher Sex: 3,6 % (95 % KI 0,3–7,5); Gewalt gegen Kinder: 2,0 % (95 % KI: 4,2–8,2).
Campbell et al. (2021) [10]	322	T1 02/2020–02/2021	Internationale Querschnittsstudie Teilauswertung der deutschen Stichprobe Retrospektive Erfassung von Gewalt vor und nach Pandemiebeginn	Deutschland Erwachsene in Partnerschaft ab 18 Jahren	Partnerschaftsgewalt: selbstkonstruierte Items	Prävalenzen: körperliche Gewalt 1,6 % vor Pandemiebeginn; keine Veränderung der Prävalenz während der Pandemie; sexuelle Gewalt/Nötigung: vor der Pandemie 0,9 %; Anstieg während der Pandemie um 1,2 %.
Mojahed et al. (2023) [12]	1054	T1 05/2020, T2 10/2020	Längsschnittliche Studie Retrospektive Erfassung von Gewalt zu 10/2020	Deutschland (Werdende) Mütter und Väter sowie deren PartnerInnen	Psychische, körperliche, sexualisierte Partnerschaftsgewalt: Revised Conflict Tactics Scale Short Version; selbstkonstruierte Items	Prävalenzen: 48,5 % psychische Gewalt an Frauen, 38,3 % psychische Gewalt an Männern; 2,6 % körperliche Gewalt an Frauen, 3,3 % körperliche Gewalt an Männern; 2,8 % sexualisierte Gewalt an Frauen, 1,5 % sexualisierte Gewalt an Männern. Im Vergleich zu vor der Pandemie 27 % Zunahme der psychischen und physischen Gewalt an Frauen, 24 % Zunahme der psychischen und 13 % Zunahme der physischen Gewalt an Männern während der Pandemie im Vergleich zu vor der Pandemie. Ein höheres Ausmaß an eigener Aggressivität zu T1 war signifikant mit einem erhöhten Risiko für das Erleben von Partnerschaftsgewalt zu T2 assoziiert. Ein jüngeres Alter, eine höhere Partnerschaftszufriedenheit sowie stärker ausgeprägte depressive Symptome waren mit einem signifikant geringeren Risiko für Partnerschaftsgewalt verbunden.
Kliem et al. (2023) [13]	4952	T1 2016, T1 2021, T1 2021	3 querschnittliche repräsentative Bevölkerungsbefragungen	Deutschland *n* = 3639 Frauen und Männer in Partnerschaft *n* = 1313 Elternteile oder Elternpaare	Körperliche und sexualisierte Partnerschaftsgewalt, körperliche und psychische Gewalt gegenüber Kindern; Module des Family Maltreatment Measure	12-Monats-Prävalenz im Jahr 2021: körperliche Gewalt gegenüber Frauen 6,4 %, gegenüber Männern 7,0 %; sexualisierte Gewalt gegenüber Frauen 3,2 %, gegenüber Männern 0,1 % Körperliche Gewalt von Müttern gegenüber jüngstem Kind 13,6 %, Väter gegenüber jüngstem Kind 14,8 %; psychische Gewalt von Müttern gegenüber jüngstem Kind 6,6 %, Väter gegenüber jüngstem Kind 5,9 %. Kein signifikanter Anstieg von körperlicher oder sexualisierter Gewalt in Partnerschaften während der COVID-19-Pandemie im Vergleich zu 2016; kein Anstieg von körperlicher oder psychischer Gewalt gegenüber dem jüngsten Kind im Vergleich zu 2016.
**Studienbefunde zu psychischen Belastungen bei traumatisch vorbelasteten Personengruppen**						
Seitz et al. (2021) [14]	85	T1 09/2018–11/2019, T2 04–05/2020	Längsschnittliche Studie Retrospektive Erfassung von Kindheitstraumata zu T1	Deutschland Frauen (78,8 %) und Männer (21,2 %) Alter 18–59 Jahre (*M* = 31,3, *SD* = 11,1) *n* = 63 Erwachsene mit psychischer Erkrankung *n* = 22 Erwachsene ohne psychische Erkrankung	Kindheitstraumata: Childhood Trauma Questionnaire Psychische Belastung: Brief Symptom Inventory; PTBS-Symptome: PTSD Checklist für DSM‑5 Soziale Unterstützung: ENRICHD Social Support Inventory	Prävalenzen: Erwachsene mit psychischer Erkrankung: 54,0 % emotionale Gewalt in der Kindheit, 27,0 % körperliche Gewalt, 28,6 % sexuelle Gewalt; 47,6 % emotionale Vernachlässigung, 30,2 % körperliche Vernachlässigung. Gesunde Erwachsene: 40,9 % emotionale Gewalt in der Kindheit, 31,8 % körperliche Gewalt, 22,7 % sexuelle Gewalt, 40,9 % emotionale Vernachlässigung, 22,7 % körperliche Vernachlässigung. Signifikante Zunahme der psychischen Belastung und PTBS-Symptome während der COVID-19-Pandemie (*ω*^*2*^ = 0,07–0,08); signifikanter Zusammenhang zwischen Schwere der Kindheitstraumata und Schwere der PTBS-Symptome (*β* = 0,25, *p* = 0,042); dieser Effekt wurde mediiert durch das Ausmaß der erlebten sozialen Unterstützung (*β* = 0,10, *SE* = 0,50).
Holl (2023) [16]	6451	T1 08/2020–02/2021	Querschnittliche Studie Retrospektive Erfassung von Kindheitstraumata	Deutschland Frauen (69,1 %), Männer (30,3 %) und 0,01 % diverse Erwachsene Alter 18–81 Jahre (*M* = 44,1, *SD* = 11,8)	Kindheitstraumata: Childhood Trauma Questionnaire; Psychische Belastung: Depression (PHQ-9), Angst (GAD-7); Persönlichkeitsmerkmale: Persönlichkeitsinventar für DSM‑5; Mentalisierung: Mentalization Scale; Emotionsdysregulation: Difficulties in Emotions Regulation Scale	Prävalenzen: psychische Gewalt: 8,2 %, körperliche Gewalt: 18,7 %, sexuelle Gewalt: 11,4 %, emotionale Vernachlässigung: 51,3 %, körperliche Vernachlässigung: 1,4 %; signifikante Zusammenhänge zwischen der Schwere verschiedener Formen von Kindheitstraumata und maladaptiven Persönlichkeitsmerkmalen. Die Beziehungen zwischen verschiedenen Formen von Kindheitstraumata und maladaptiven Persönlichkeitsmerkmalen bzw. Depressions- und Angstsymptomen wurden sowohl durch die Mentalisierungsfähigkeit als auch durch die emotionsregulativen Fähigkeiten mediiert.
Flechsenhar und Bertsch (2023) [18]	116	T1 04/2020, T2 07/2020, T3 01/2021	Längsschnittliche Studie Retrospektive Erfassung von Lebenszeittraumata zu T1	Deutschland Erwachsene ab 18 Jahren Alter 22–84 Jahre (*M* = 60,7, *SD* = 16,8), 53,4 % weiblich	Lebenszeittraumata: Brief-Trauma Questionnaire Psychische Belastung: Brief Symptom Inventory; Depression: Brief Depression Inventory	Prävalenzen: 40,4 % mind. ein erlebtes Lebenszeittrauma; 32,6 % Kindheitstraumata (19,5 % körperliche Gewalt, 15,2 % sexuelle Gewalt in der Kindheit). Erlebte Traumata zeigten keinen signifikanten Einfluss auf die psychische Belastung oder Depression (*p* = 0,08); deskriptiv erhöhten sich die Depressionssymptome jedoch bei Teilnehmenden mit Traumaerfahrung über alle 3 Erhebungszeitpunkte hinweg. BDI: T1 *M* = 5,5, *SD* = 6,2; T2 *M* = 6,0, *SD* = 7,8; T3 *M* = 6,5, *SD* = 6,6.
Lotzin et al. (2022) [19, 20]	2744	T1 06–09/2020	Teilauswertung der deutschen Daten einer querschnittlichen Studie Erfassung von Traumata während und vor der Pandemie	Deutschland Alter 18–89 Jahre Alter *M* = 43,8, *SD* = 14,4 73,0 % weiblich	PTBS-Symptome: Primary Care Checklist for PTSD Traumata: Life Events Checkliste der PTSD Checklist für DSM‑5 Pandemiebezogene Stressoren: Pandemic Stressor Scale	Prävalenzen: 87,6 % mind. ein erlebtes Trauma vor Pandemiebeginn, 23,2 % nach Pandemiebeginn; PTBS 17,7 %. Signifikante Zusammenhänge mit erhöhtem PTBS-Risiko (*p* < 0,001): weibliches Geschlecht, jüngeres Alter, erhöhtes Risiko für einen schweren COVID-19-Verlauf, aktuelle oder frühere Diagnose einer psychischen Erkrankung, unzureichende Kommunikation und unzureichendes Krisenmanagement der Regierung, schwierige Wohnverhältnisse, arbeitsbezogene Probleme, eingeschränkte Sozialkontakte. Signifikante Zusammenhänge mit geringerem PTBS-Risiko (*p* < 0,001): Sozialkontakt, sehr guter Gesundheitszustand.
Lueger-Schuster et al. (2022) [21]	243	T1 06/2020–12/2021	Längsschnittliche Studie 4 Erhebungszeitpunkte alle 6 Monate Erfassung von PTBS-Symptomen in verschiedenen Phasen der Pandemie	Österreich Erwachsene Alter 21–81 Jahre (*M* = 48,6, *SD* = 15,0) 67,5 % weiblich	PTBS: Primary Care PTSD Screen for DSM‑5	Prävalenzen: PTBS T1 7,7 %, T2 0,5 %, T3 2,3 %, T4 4,5 %. PTBS-Symptomschwere: T1 *M* = 0,94, *SD* = 1,37; T2 *M* = 1,37, *SD* = 1,24; T3 *M* = 1,30, *SD* = 1,47; T4 *M* = 1,57, *SD* = 1,81.

*Abkürzungen.* *SD* Standardabweichung, *M* Mittelwert, *BDI* Beck-Depressions-Inventar, *DSM‑5* 5. Auflage des Diagnostic and Statistical Manual of Mental Disorders, *GAD‑7* Generalized Anxiety Disorder Scale‑7, *KI* Konfidenzintervall, *PHQ‑9* Patient Health Questionnaire‑9, *PTBS* posttraumatische Belastungsstörung, *PTSD* „post-traumatic stress disorder“, *T1* Messzeitpunkt 1, *T2* Messzeitpunkt 2, *T3* Messzeitpunkt 3, *WHO* Weltgesundheitsorganisation

### Häusliche Gewalt in der frühen Phase der COVID-19-Pandemie.

In einer deutschen Repräsentativbefragung von *N* = 3818 Frauen in Partnerschaften in der frühen Phase der Pandemie (April bis Mai 2020) wurden körperliche und psychische Gewalt durch den Partner oder die Partnerin im letzten Monat erfasst [9]. Es wurden verschiedene Formen psychischer Partnerschaftsgewalt sowie leichtere Formen körperlicher Gewalt per Fragebogen erhoben, während sexualisierte Gewalt und schwere Formen körperlicher Gewalt indirekt durch ein Listenexperiment erfasst wurden. Es berichteten 3,1 % der Frauen, körperliche Gewalt innerhalb des letzten Monats erfahren zu haben; 3,6 % berichteten sexualisierte Gewalt und 7,7 % berichteten psychische Gewalt durch den Partner oder die Partnerin. Körperliche Gewalt gegenüber ihren Kindern im letzten Monat berichteten 6,6 % der befragten Frauen. Zum Erhebungszeitpunkt waren in Deutschland seit mehr als einem Monat starke Lockdown-Beschränkungen eingeführt. Ein erhöhtes Risiko für das Erleben körperlicher Partnerschaftsgewalt bestand bei häuslicher Quarantäne, finanziellen Sorgen, schlechterer psychischer Gesundheit sowie bei Vorhandensein jüngerer Kinder im Haushalt. Ähnliche Risikofaktoren zeigten sich für sexualisierte und psychische Gewalt. Der Bekanntheitsgrad und die Inanspruchnahme von Unterstützungsangeboten waren bei den von Gewalt Betroffenen gering.

### Partnerschaftsgewalt in der frühen Phase der COVID-19-Pandemie.

In einer internationalen Studie zur sexuellen und reproduktiven Gesundheit während der Pandemie (I-SHARE^1^; [10, 11]) wurden in einer deutschen querschnittlichen Teilstudie bei *N* = 322 in Partnerschaften lebenden Erwachsenen Partnerschaftsgewalt untersucht. Angaben zu den ersten 3 Monaten der COVID-19-Pandemie wurden mit retrospektiven Angaben zu den letzten 3 Monaten vor der Pandemie verglichen. Die Stichprobe umfasste überwiegend weibliche (83,2 %, *n* = 268), jüngere (*M* = 29,2, *SD* = 9,2 Jahre) Teilnehmende mit hohem Bildungsstand. Von den Teilnehmenden gaben 1,6 % an, vor Beginn der Pandemie körperliche Gewalt in ihrer Partnerschaft erlebt zu haben. Zu Beginn der Pandemie nahm die Häufigkeit weder zu noch ab. Hingegen gaben 0,9 % der Teilnehmenden an, vor Beginn der Pandemie sexualisierte Gewalt oder Nötigung in ihrer Partnerschaft erlebt zu haben; zu Beginn der Pandemie stieg der Anteil auf 1,2 % an. Das Erleben von Partnerschaftsgewalt ging mit einer erhöhten psychischen Belastung einher.

### Partnerschaftsgewalt bei (werdenden) Eltern im ersten Jahr der COVID-19-Pandemie.

In einer deutschen Teilstudie zu Partnerschaftsgewalt bei (werdenden) Eltern während der COVID-19-Pandemie (DREAMCORONA^2^; [12]) wurden Daten von *N* = 1054 (werdenden) Müttern und Vätern sowie ihren Partnerinnen oder Partnern aus der Allgemeinbevölkerung mittels Onlinesurvey im Mai und Oktober 2020 erhoben. Art und Ausmaß der Partnerschaftsgewalt wurden mit der Kurzform der Revised Conflict Tactic Scale zum zweiten Messzeitpunkt bewertet. Es wurden emotionale, körperliche und sexualisierte Partnerschaftsgewalt erfasst. Zusätzlich wurde retrospektiv erfragt, ob sich die Häufigkeit der Partnerschaftsgewalt während der Pandemie im Vergleich zu vor der Pandemie verändert habe. Frauen und Männer berichteten, am häufigsten psychischer Partnerschaftsgewalt ausgesetzt gewesen zu sein (Frauen 48,5 %; Männer 38,3 %). Die Betroffenheit von körperlicher Gewalt wurde von 2,6 % der Frauen und 3,3 % der Männer angegeben, sexualisierte Gewalt wurde von 2,8 % der Frauen und 1,5 % der Männer berichtet. Die Häufigkeit der verschiedenen Formen von Partnerschaftsgewalt während der Pandemie im Vergleich zu vor der Pandemie wurde von der Mehrheit der Befragten als unverändert beschrieben. Etwa 27 % der Frauen mit Gewalterfahrungen gaben jedoch an, dass psychische und physische Gewalt zugenommen hatten. Dies traf ebenfalls auf 13–24 % der Männer zu. Ein höheres Ausmaß an eigener Aggressivität und Feindseligkeit, erhoben zum ersten Messzeitpunkt, war mit einem erhöhten Risiko für das Erleben von Partnerschaftsgewalt assoziiert. Ein jüngeres Alter, eine höhere Partnerschaftszufriedenheit sowie stärker ausgeprägte depressive Symptome waren hingegen mit einem geringeren Risiko für Partnerschaftsgewalt verbunden.

### Häusliche Gewalt im zweiten Jahr der COVID-19-Pandemie.

In 3 repräsentativen Bevölkerungsbefragungen (DoViCov^3^-Studie; [13]) wurden die Prävalenzen verschiedener Formen häuslicher Gewalt (körperliche und sexualisierte Partnerschaftsgewalt, körperliche und psychische Gewalt gegenüber Kindern) mit dem Family Maltreatment Measure bei *N* = 3639 Frauen und Männern in Partnerschaften sowie bei *N* = 1313 Eltern in Deutschland untersucht. Zur Analyse eines möglichen Anstiegs häuslicher Gewalt wurden die Befragungsdaten vor der COVID-19-Pandemie (Jahr 2016) mit den Daten einer weiteren Befragung im zweiten Jahr der Pandemie (Jahr 2021) verglichen. Im Jahr 2021 betrugen die 12-Monats-Prävalenzen für körperliche Partnerschaftsgewalt gegenüber Frauen 6,4 %, gegenüber Männern 7,0 %. Sexualisierte Partnerschaftsgewalt wurde von 3,2 % der weiblichen und 0,1 % der männlichen Opfer berichtet. Die 12-Monats-Prävalenz für körperliche Gewalt von Müttern gegenüber dem jüngsten Kind betrug 13,6 %, von Vätern gegenüber dem jüngsten Kind lag sie mit 14,8 % geringfügig höher. Psychische Gewalt gegenüber Kindern wurde von 6,6 % der Mütter und 5,9 % der Väter ausgeübt. Im Vergleich zu den Daten vor der COVID-19-Pandemie wurde kein signifikanter Anstieg von körperlicher, psychischer oder sexualisierter Gewalt in Partnerschaften oder gegenüber Kindern festgestellt.

## Studienbefunde zu psychischen Belastungen bei traumatisch vorbelasteten Personengruppen

Zum Themenbereich psychische Belastungen bei Risikogruppen wurden 5 Studien bzw. Sekundäranalysen in die Übersicht einbezogen (Tab. 1), die im Folgenden beschrieben werden.

### Psychische Belastung und Symptome der posttraumatischen Belastungsstörung (PTBS) bei Erwachsenen in der frühen Phase der COVID-19-Pandemie.

In einer längsschnittlichen deutschen Teilstudie [14] einer größeren Studie (Graduiertenkolleg 2350, Deutsche Forschungsgemeinschaft, Projekt C1) wurden *N* = 85 weibliche Erwachsene mit überwiegend psychischen Erkrankungen und hohem Bildungsstand retrospektiv zu eigenen Kindheitstraumata (emotionale, körperliche und sexualisierte Gewalt, emotionale und körperliche Vernachlässigung) mit dem Childhood Trauma Questionnaire (CTQ) befragt. Zusätzlich wurden PTBS-Symptome und die allgemeine psychische Belastung vor der Pandemie (September 2018 bis November 2019) sowie zu Beginn der Pandemie (April bis Mai 2020) erhoben. Unter Berücksichtigung der Cut-off-Werte für mäßig bis schwere Kindheitstraumata laut CTQ-Subskalen [15] berichteten in der Gruppe der Erwachsenen mit psychischen Erkrankungen (*n* = 63; 74,1 %) 54,0 % emotionale Gewalt, 27,0 % körperliche Gewalt und 28,6 % sexualisierte Gewalt; 47,6 % berichteten emotionale Vernachlässigung und 30,2 % körperliche Vernachlässigung. Bei den Erwachsenen ohne psychische Erkrankungen lagen die Prävalenzraten niedriger: emotionale Gewalt (40,9 %), sexualisierte Gewalt (22,7 %), emotionale Vernachlässigung (40,9 %), körperliche Vernachlässigung (22,7 %); nur bei körperlicher Gewalt lagen sie mit 31,8 % etwas höher. In beiden untersuchten Gruppen nahmen die PTBS-Symptome und die psychische Belastung während der Pandemie im Vergleich zu vor der Pandemie zu. Mit der Schwere der Kindheitstraumata ging eine Zunahme der PTBS-Symptome während der Pandemie einher. Das Ausmaß der erlebten sozialen Unterstützung mediierte den Zusammenhang zwischen Kindheitstraumata und der Zunahme posttraumatischer Belastungssymptome.

### Allgemeine psychische Belastung bei Erwachsenen mit Kindheitstraumata während der COVID-19-Pandemie.

In einer querschnittlichen Analyse [16] einer längsschnittlichen deutschen Studie (PACE^4^-Studie; [17]) wurden *N* = 6451 Erwachsene (Alter *M* = 44,1, *SD* = 11,8; 69,1 % Frauen, 30,3 % Männer, 0,01 % divers mit hohem Bildungsstand) während der COVID-19-Pandemie retrospektiv zu eigenen Kindheitstraumata mit dem Childhood Trauma Questionnaire befragt sowie zu Depressions- und Angstsymptomen, maladaptiven Persönlichkeitsmerkmalen, Emotionsdysregulation und Mentalisierungsfähigkeit. Die Datenerhebung fand von August 2020 bis Februar 2021 statt. Es ergaben sich folgende Prävalenzen für Kindheitstraumata (mindestens mittlerer Schweregrad): psychische Gewalt 8,2 %, körperliche Gewalt 18,7 %, sexualisierte Gewalt 11,4 %, emotionale Vernachlässigung 51,3 % sowie körperliche Vernachlässigung 1,4 %. Zudem zeigten sich signifikante Zusammenhänge zwischen verschiedenen Formen von Kindheitstraumata und maladaptiven Persönlichkeitsmerkmalen. Die Beziehungen zwischen verschiedenen Formen von Kindheitstraumata und maladaptiven Persönlichkeitsmerkmalen bzw. Depressions- und Angstsymptomen wurden sowohl durch die Mentalisierungsfähigkeit als auch durch die emotionsregulativen Fähigkeiten mediiert.

### Psychische Belastung und depressive Symptome während der COVID-19-Pandemie bei Erwachsenen mit Lebenszeittraumata.

Die längsschnittliche Studie [18] untersuchte *N* = 116 gesunde, überwiegend ältere Erwachsene (Alter: *M* = 60,7, *SD* = 16,8 Jahre, Spanne 22–84 Jahre, weiblich: 53,4 %) während der COVID-19-Pandemie in Deutschland. Es wurden Zusammenhänge zwischen Lebenszeittraumata (gemessen mittels Brief-Trauma Questionnaire – BTQ), der allgemeinen psychischen Belastung und depressiven Symptomen ermittelt. Die Befragung wurde online oder postalisch zu 3 Zeitpunkten (April 2020, Juli 2020, Januar 2021) durchgeführt, die durch ein unterschiedliches Maß an Infektionsschutzmaßnahmen gekennzeichnet waren. Es berichteten 40,4 % der Befragten mindestens eine erlebte Traumatisierung während der Lebenszeit. Von den 32,6 %, die ein Kindheitstrauma angaben, berichteten 19,5 % von körperlicher Gewalt und 15,2 % von sexualisierter Gewalt. Eine Traumatisierung während der Lebenszeit hatte keinen signifikanten Einfluss auf die psychische Belastung oder depressive Stimmung während der Pandemie.

### PTBS-Risiko während der COVID-19-Pandemie bei Erwachsenen mit Lebenszeittraumata.

In einer querschnittlichen Teilstudie einer internationalen Kohortenstudie (ESTSS ADJUST-Studie^5^; [19, 20]) wurden Erwachsene mit Lebenszeittraumata in Deutschland (*N* = 2744) von Juni bis September 2020 zu Risikofaktoren einer PTBS befragt. Faktoren, die mit einem erhöhten PTBS-Risiko einhergingen, betrafen ein weibliches Geschlecht, ein jüngeres Alter, ein erhöhtes Risiko für einen schweren Verlauf einer COVID-19-Erkrankung sowie eine aktuelle oder frühere Diagnose einer psychischen Erkrankung [19]. Eine unzureichende Kommunikation über die Pandemie und entsprechende Maßnahmen sowie ein unzureichendes Krisenmanagement der Regierung, schwierige Wohnverhältnisse, arbeitsbezogene Probleme und eingeschränkte Sozialkontakte waren ebenfalls mit einem erhöhten PTBS-Risiko verbunden. Häufigerer Sozialkontakt und ein sehr guter Gesundheitszustand waren dagegen mit einem verringerten PTBS-Risiko assoziiert.

### Verlauf von PTBS-Symptomen während der COVID-19-Pandemie.

Eine österreichische längsschnittliche Teilstudie [21] einer internationalen Kohortenstudie (ESTSS ADJUST-Studie; [20]) untersuchte den Verlauf von PTBS-Symptomen in der Allgemeinbevölkerung. Insgesamt wurden *N* = 243 österreichische Erwachsene zu 4 Zeitpunkten zwischen Juni 2020 und Dezember 2021 mittels Onlinebefragung untersucht. Zwischen 0,5 % und 7,7 % der Befragten berichteten erhöhte PTBS-Symptome. Mit Fortschreiten der Pandemie fand sich eine Zunahme an PTBS-Symptomen. Zudem zeigte sich ein saisonaler Trend: In den Wintermonaten nahmen die PTBS-Symptome im Vergleich zu den Sommermonaten zu. Die Wintermonate zeichneten sich im Vergleich zu den Sommermonaten durch höhere COVID-19-bezogene Erkrankungs- und Todesraten sowie strengere Präventionsmaßnahmen aus. Darüber hinaus berichteten die Befragten in den Wintermonaten eine Abnahme gesundheitsförderlicher sowie angenehmer Aktivitäten.

## Diskussion

Ziel dieses Beitrags war es, zentrale Studienbefunde zur Prävalenz von häuslicher Gewalt und damit einhergehende psychische Belastungen während der COVID-19-Pandemie im deutschsprachigen Raum zusammenzufassen. Darüber hinaus sollten Zusammenhänge zwischen Kindheitstraumata und aktuellen psychischen Belastungen während der Pandemie dargestellt werden.

### Häusliche Gewalt während der COVID-19-Pandemie

Insgesamt sprechen die Befunde der im deutschsprachigen Raum durchgeführten Studien für hohe Prävalenzen häuslicher körperlicher Gewalt (2–18 %; [9, 10, 12, 13]) und sexualisierter Gewalt (1–8 %; [9, 10, 12, 13]). Bezüglich psychischer Partnerschaftsgewalt divergierten die Prävalenzen in verschiedenen Studien deutlich [9, 12]. Die 3‑Monats-Prävalenz für psychische Partnerschaftsgewalt betrug in einer deutschen Repräsentativbefragung von Frauen 8 % [9]. Eine deutsche Studie an (werdenden) Müttern und Vätern berichtete hingegen deutlich höhere 12-Monats-Prävalenzen von 49 % gegenüber Frauen und 38 % gegenüber Männern [12]. Hierbei ist zu berücksichtigen, dass die durchgeführten Studien unterschiedliche Erhebungsinstrumente und Schwellenwerte für das Vorliegen psychischer Gewalt verwendeten, die zu Unterschieden in den Prävalenzen beitragen können. Hierdurch ist die Vergleichbarkeit der Studienergebnisse aus verschiedenen Studien eingeschränkt.

Häufig diskutiert wurde in Fachkreisen die Frage, ob während der Pandemie ein Anstieg an häuslicher Gewalt erfolgt ist. 3 der hier vorgestellten Studien liefern hierzu Daten, die zu unterschiedlichen Zeitpunkten der Pandemie in Deutschland erhoben worden sind [10, 12, 13]. Auf der Basis repräsentativer Bevölkerungsdaten vor und während der COVID-19-Pandemie (DoViCov-Studie; [13]) zeigte sich bezüglich körperlicher Gewalt gegenüber weiblichen Partnerinnen und männlichen Partnern kein Anstieg ebenso wenig gegenüber Kindern. In 2 weiteren in Deutschland durchgeführten retrospektiven Befragungen war ebenfalls kein signifikanter Anstieg von körperlicher häuslicher Gewalt in der Allgemeinbevölkerung erkennbar (Subgruppenanalyse zu [10, 12]).

In den vorliegenden deutschen Repräsentativdaten zu sexualisierter Gewalt gegenüber der Partnerin bzw. dem Partner bildete sich im Vergleich zum Zeitraum vor der Pandemie keine Veränderung ab [13]; hingegen berichtete eine nicht-repräsentative deutsche Studie einen leichten Anstieg an sexualisierter Partnerschaftsgewalt (Subgruppenanalyse [10]). In einer weiteren Studie zu sexualisierter Gewalt bei Eltern gaben sowohl Mütter als auch Väter keinen Anstieg während der Pandemie an [12]. Bezüglich psychischer Gewalt gab die Mehrheit der viktimisierten (werdenden) Mütter und Väter an, dass sich die Häufigkeit der psychischen Gewalt während der Pandemie nicht verändert hatte [12]; ein Viertel der (werdenden) Mütter (27 %) und Väter (13–24 %) berichtete hingegen eine Zunahme psychischer und körperlicher Partnerschaftsgewalt.

Insgesamt bestätigen die vorliegenden Befunde auf der Basis von Befragungsdaten im deutschsprachigen Raum hohe Prävalenzen für häusliche Gewalt während der COVID-19-Pandemie, die weitgehend den Prävalenzen vor der Pandemie entsprechen. 2 der 4 einbezogenen Studien bzw. Sekundäranalysen zu häuslicher Gewalt basieren jedoch auf nicht-repräsentativen Daten. Hierdurch können die berichteten Prävalenzen sowohl über- als auch unterschätzt werden. Darüber hinaus können retrospektive Einschätzungen der Prävalenzen häuslicher Gewalt vor der Pandemie Erinnerungseffekten unterliegen. Unterschiedliche Schwellenwerte der eingesetzten Fragebögen für verschiedene Formen von Gewalt gehen mit unterschiedlichen Prävalenzen einher, sodass sich Veränderungen in den Prävalenzen nur in längsschnittlichen Studien unter Verwendung desselben Messinstruments abschätzen lassen. Darüber hinaus erfasste ein Teil der Studien zwar, ob verschiedene Formen von Gewalt aufgetreten sind, Veränderungen in der Häufigkeit des Erlebens von Gewalt wurden jedoch nicht immer berücksichtigt.

Weiter gilt es zu bedenken, dass auch Repräsentativbefragungen Selektionseffekten unterliegen, etwa weil nicht alle angefragten Personen bereit sind, an der Studie teilzunehmen oder im Verlauf der Studie nicht weiter teilnehmen möchten. Insbesondere hoch belastete Personen, die von schweren Formen körperlicher und sexualisierter Gewalt betroffen sind, könnten weniger Ressourcen haben, an Studien teilzunehmen. Schließlich ist darauf hinzuweisen, dass die Prävalenzen im Mittel in den untersuchten Gruppen nicht signifikant anstiegen; es ist jedoch davon auszugehen, dass in Subgruppen ein Anstieg oder eine Abnahme von Gewalt seit der Pandemie zu verzeichnen ist [12].

Die international vorliegenden Befunde zur Prävalenz von häuslicher Gewalt während der COVID-19-Pandemie fallen inkonsistent aus [22]. Eine Metaanalyse von Hellfelddaten (z. B. Kriminalstatistiken, Krankenhausdaten) aus dem überwiegend US-amerikanischen Raum ergab eine Zunahme häuslicher Gewalt um 8 % nach Einführung der Infektionsschutzmaßnahmen [23]. Bezüglich körperlicher Gewalt gegenüber Kindern wurde in internationalen Studien ein Rückgang von Gewalt während der Pandemie berichtet [24, 25]. Eine australische retrospektive Befragung berichtete einen Anstieg sexualisierter Partnerschaftsgewalt während der frühen Phase der Pandemie [26]. Die meisten dieser Untersuchungen, die eine Zunahme an häuslicher Gewalt berichten, basieren jedoch auf Hellfelddaten, die durch die Offenbarungs- und Anzeigebereitschaft sowie durch das Hilfesuchverhalten der Betroffenen beeinflusst werden. So kann ein Anstieg polizeilicher Anzeigen häuslicher Gewalt auf eine tatsächliche Zunahme hinweisen, jedoch auch auf eine gesteigerte Sensibilität der Bevölkerung für häusliche Gewalt. Unabhängig davon, ob die bereits vor der Pandemie hohen Prävalenzzahlen häuslicher Gewalt durch die Infektionsschutzmaßnahmen weiter angestiegen sind, ist zusätzlich zu den berichteten Prävalenzen häuslicher Gewalt ein großes Dunkelfeld anzunehmen.

Insgesamt sind nur wenige repräsentative Studiendaten zu Gewalt und anderen Traumata im deutschsprachigen Raum sowie international verfügbar. Die vorliegenden Befunde sollten anhand weiterer repräsentativer Studiendaten verifiziert werden, um die methodischen Einschränkungen der bisherigen Studien auszugleichen. Darüber hinaus sollten Risikofaktoren für bestimmte Formen häuslicher Gewalt identifiziert werden, um Interventionen für individualisierte Präventionsansätze zu entwickeln. Dazu zählen z. B. vorbestehende Gewalterfahrungen, Substanzkonsum, eine bestehende psychische Erkrankung und finanzielle Belastungen. Um das Zustandekommen heterogener Studienergebnisse besser erklären zu können, sollten künftige Studien moderierende Faktoren berücksichtigen, wie zum Beispiel das Ausmaß des Einsatzes von Infektionsschutzmaßnahmen und der sozialen Unterstützung. Weitreichendere Infektionsschutzmaßnahmen, wie z. B. Kontakt- und Ausgangsbeschränkungen, könnten mit einer erhöhten psychischen Belastung einhergehen, welche wiederum ein Risikofaktor für häusliche Gewalt darstellt [27]. Demnach erscheint es lohnend, in zukünftigen Auswertungen von Studiendaten Zusammenhänge zwischen dem Ausmaß der Infektionsschutzmaßnahmen und der Prävalenz verschiedener Formen von Gewalt zu betrachten.

### Psychische Belastungen bei traumatisch vorbelasteten Erwachsenen

In Deutschland erkrankten vor der Pandemie ca. 12 % der von traumatischen Ereignissen (z. B. häusliche Gewalt) Betroffenen an PTBS [28]. Vor dem Hintergrund der vielfältigen Stressoren während einer Pandemie ist davon auszugehen, dass Menschen mit traumatischen Vorbelastungen ein erhöhtes Risiko für die Erstmanifestation oder Symptomverschlechterung posttraumatischer Belastungsstörungen aufweisen im Vergleich zu vor der Pandemie. Sowohl in Deutschland als auch international liegen nur wenige Befunde zu Risikofaktoren posttraumatischer Belastungsstörungen während der COVID-19-Pandemie vor. Eine Ausnahme bildet eine Teilauswertung von deutschen Studiendaten einer internationalen Studie zu Risiko- und Schutzfaktoren einer PTBS bei Erwachsenen, die verschiedene Formen traumatischer Erfahrungen vor oder während der COVID-19-Pandemie erfahren hatten [19]. Es konnte eine Reihe an Faktoren bzw. Stressoren identifiziert werden, die das Risiko einer PTBS während der COVID-19-Pandemie erhöht haben: biologische (z. B. jüngeres Alter, weibliches Geschlecht), psychologische (z. B. aktuelle oder frühere Diagnose einer psychischen Erkrankung) und soziale (z. B. schwierige Wohnverhältnisse). Darüber hinaus konnten Faktoren ausgemacht werden, die mit einem geringeren Risiko einer PTBS einhergingen, wie z. B. ein häufigerer Sozialkontakt und ein sehr guter Gesundheitszustand.

Diese im deutschsprachigen Raum gefundenen Ergebnisse decken sich mit internationalen Befunden, die als Risikofaktoren für erhöhte PTBS-Symptome ein jüngeres Alter [29], ein weibliches Geschlecht [29, 30], seltenere Sozialkontakte [30] und einen schlechteren allgemeinen Gesundheitszustand [29] ausmachten. Die Identifizierung von Risiko- und Schutzfaktoren für die Entwicklung posttraumatischer Belastungsstörungen im Rahmen der aktuellen COVID-19-Pandemie könnte einen Beitrag dazu leisten, sowohl die subjektiven Beschwerden der Betroffenen zu reduzieren als auch die Gesundheitssysteme im Bereich der Akutversorgung zu entlasten, wenn rechtzeitig psychosoziale Unterstützungsangebote zur Verfügung gestellt werden. Hierbei könnten spezifisch solche Bereiche adressiert werden, die Zusammenhänge mit PTBS-Symptomen bzw. psychischer Belastung gezeigt haben, wie z. B. geringe soziale Unterstützung oder Vorbelastungen durch eine frühere psychische Erkrankung.

Für den österreichischen Raum liegen Befunde über den zeitlichen Verlauf von PTBS-Symptomen im Zuge der COVID-19-Pandemie vor [21]. Hier zeigte sich eine steigende Tendenz innerhalb eines 18-monatigen Zeitraums von Sommer 2020 bis Winter 2021. Studiendaten aus anderen Ländern zeigten nicht immer einen Zusammenhang zwischen der Chronizität der Pandemie und einer Zunahme an PTBS-Symptomen. In Italien zeigte sich ein Anstieg der PTBS-Symptome im Herbst 2020, jedoch nicht im Frühling 2020 [31]; in einer spanischen Studie fanden sich keine Veränderungen im Sinne einer Zu- oder Abnahme im Verlauf des ersten Pandemiejahres 2020 [32]. In einer repräsentativen Studie in Deutschland, Polen, Slowenien und Israel an jungen Erwachsenen im zweiten Pandemiejahr 2021 deuten die Daten im Verlauf von Januar bis Juni 2021 auf einen geringfügigen Rückgang der Symptomatik hin [33]. Es ist anzunehmen, dass unterschiedliche PTBS-Symptomverläufe mit der Intensität der Infektionswellen, Infektionsschutzmaßnahmen, jahreszeitlichen Veränderungen und dem Alter der Teilnehmenden zusammenhängen. Weitere längsschnittliche Datenanalysen sind notwendig, um Symptomverläufe in verschiedenen Pandemiephasen der Pandemie sowie dahinterliegende Mechanismen besser zu verstehen.

Bei Erwachsenen mit traumatischen Kindheitserfahrungen wurden in 2 deutschen Studien klinische Symptome während der COVID-19-Pandemie im Vergleich zu vor der Pandemie untersucht [14, 16]. Betroffene berichteten einen Anstieg der allgemeinen psychischen Belastung, der PTBS-Symptome [14] und der Angst- und Depressionssymptome [16] in der frühen Phase der Pandemie im Vergleich zu vor der Pandemie. Die Ergebnisse der längsschnittlichen Studie [14] stützen die Annahme, dass traumatische Kindheitserfahrungen einen Risikofaktor für einen Anstieg an psychischer Belastung bzw. PTBS-Symptomen während der COVID-19-Pandemie darstellen. Ähnliche Befunde wurden in internationalen Studien berichtet [5, 34, 35]. Eine der deutschen Studien [16] stützt bisherige internationale Befunde, die Zusammenhänge zwischen traumatischen Kindheitserfahrungen, Emotionsregulation, Mentalisierungsfähigkeit und erhöhter psychischer Belastung berichtet haben [36, 37]. Diese Zusammenhänge könnten darauf hinweisen, dass Personen mit Gewalterfahrungen in der Kindheit eine geringere Emotionsregulation und Mentalisierungsfähigkeit aufweisen, die die Bewältigung pandemiebedingter Stressoren erschwert und hierdurch die psychische Belastung erhöht. Entsprechende kausale Zusammenhänge müssten jedoch in längsschnittlichen oder experimentellen Studien nachgewiesen werden. In der weiteren deutschen Studie [14] wurde – konsistent zu früheren Befunden – [38] berichtet, dass ein höheres Ausmaß an erlebter sozialer Unterstützung mit geringeren PTBS-Symptomen zusammenhing. Weiter zeigte sich, dass das Ausmaß sozialer Unterstützung den Zusammenhang zwischen Kindheitstraumata und posttraumatischen Belastungssymptomen mediierte. Nach diesen Ergebnissen könnte soziale Unterstützung dazu beitragen, posttraumatische Belastungssymptome in pandemischen Kontexten zu reduzieren.

## Fazit

Der allgemein hohen Prävalenz häuslicher Gewalt sollte während einer Pandemie – aber auch außerhalb davon – Rechnung getragen werden. Häusliche Gewalt geht mit erheblichen körperlichen, psychischen und ökonomischen Belastungen einher. Eine stetige Öffentlichkeitsarbeit ist notwendig, um Hinweise auf häusliche Gewalt gezielt zu explorieren und Betroffene zu unterstützen. Von häuslicher Gewalt Betroffenen, die anhaltende psychische Belastung aufgrund aktueller oder früherer Gewalterfahrungen schildern, sollten psychosoziale und psychotherapeutische Unterstützungsmöglichkeiten angeboten werden. Angesichts der aktuellen globalen Krisen wären zudem breit angelegte präventive Kampagnen wichtig, um die Bewältigungskompetenz der Allgemeinbevölkerung und des Gesundheitssystems im Umgang mit häuslicher Gewalt zu erhöhen und Abwärtsspiralen zu verhindern.

## Footnotes

1 I‑SHARE: International Sexual HeAlth and REproductive Health.

2 DREAMCORONA: Teilstudie zu Corona der DResdner Studie zu Elternschaft, Arbeit und Mentaler Gesundheit.

3 DoViCov: The Effect of the Covid-19 Pandemic on Domestic Violence in Germany.

4 PACE: Psychosoziale Auswirkungen der Corona-Pandemie.

5 The European Society for Traumatic Stress Studies COVID-19 ADJUST-Study, https://estss.org/adjust.
